# Prognostic impact of changes in aortic stiffness for cardiovascular and mortality outcomes in individuals with type 2 diabetes: the Rio de Janeiro cohort study

**DOI:** 10.1186/s12933-022-01514-8

**Published:** 2022-05-14

**Authors:** Claudia R L Cardoso, Nathalie C Leite, Gil Fernando Salles

**Affiliations:** Department of Internal Medicine, School of Medicine, University Hospital Clementino Fraga Filho, Universidade Federal do Rio de Janeiro, Rio de Janeiro, Rua Croton, 72 Jacarepagua, Rio de Janeiro, RJ CEP: 22750-240 Brasil

**Keywords:** Aortic stiffness, Cardiovascular events, Carotid-femoral pulse wave velocity, Cohort study, Mortality, Type 2 diabetes

## Abstract

**Background:**

The prognostic importance of changes in aortic stiffness for the occurrence of adverse cardiovascular outcomes and mortality has never been investigated in patients with type 2 diabetes. We aimed to evaluate it in a cohort of 417 patients.

**Methods:**

Changes in aortic stiffness were assessed by 2 carotid-femoral pulse wave velocity (CF-PWV) measurements performed over a 4-year period. Multivariable Cox analysis examined the associations between changes in CF-PWV, evaluated as a continuous variable with splines and as categorical ones (quartiles and stable/reduction/increase subgroups), and the occurrence of total cardiovascular events (CVEs), major adverse CVEs (MACEs), and all-cause and cardiovascular mortality.

**Results:**

Over a median follow-up of 8.2 years after the 2nd CF-PWV measurement, there were 101 total CVEs (85 MACEs) and 135 all-cause deaths (64 cardiovascular). As a continuous variable, the lowest risk nadir was at -2.5%/year of CF-PWV change, with significantly higher risks of mortality associated with CF-PWV increases, but no excess risks at extremes of CF-PWV reduction. Otherwise, in categorical analyses, patients in the 1st quartile (greatest CF-PWV reductions) had excess risks of all-cause and cardiovascular mortality (hazard ratios [HRs]: 2.0–2.7), whereas patients in 3rd quartile had higher risks of all outcomes (HRs: 2.0–3.2), in relation to the lowest risk 2nd quartile subgroup. Patients in the 4th quartile had higher risks of all-cause mortality. Categorization as stable/reduction/increase subgroups was confirmatory, with higher risks at greater reductions (HRs: 1.7–3.3) and at greater increases in CF-PWV (HRs: 1.9–3.4), in relation to those with stable CF-PWV.

**Conclusions:**

Changes in aortic stiffness, mainly increases and possibly also extreme reductions, are predictors of adverse cardiovascular outcomes and mortality in individuals with type 2 diabetes.

## Background

Individuals with diabetes have greater arterial stiffness [1–3] and higher cardiovascular morbidity and mortality than general populations [4–6]. This higher cardiovascular risk is not completely explained by clustering of traditional risk factors and increased arterial stiffness may be one pathophysiological mechanism linking diabetes to increased cardiovascular morbidity and mortality [7]. Increased aortic stiffness, evaluated by its gold-standard carotid-femoral pulse wave velocity (CF-PWV) measurement [8], has been largely associated with adverse cardiovascular events and all-cause mortality in different populations and clinical conditions [8–10], including in diabetes [11, 12]. Because of its prognostic importance, some studies investigated the factors associated with aortic stiffness progression in diverse populations [13–17], including diabetes [18]. Ageing and high blood pressure (BP) are its main determinants, but African ancestry, abdominal obesity, higher heart rate, and worse renal function had also been related to its increase [13–17]. In type 2 diabetic individuals, female gender, higher heart rates, dyslipidemia, the presence of diabetic retinopathy and poorer glycemic control were associated with aortic stiffness progression, besides age and systolic BP (SBP) [18]. In this study, it was possible to establish temporal associations between high HbA_1c_ levels and aortic stiffness progression, which might be in part mediated by advanced glycation end-products (AGEs) formation [18].

On the other hand, some studies demonstrated that reducing aortic stiffness (‘de-stiffening’) is feasible [8], by either non-pharmacological interventions, such as weight loss [19] and regular physical activity [20], or by pharmacological interventions such as renin-angiotensin-aldosterone system inhibitors [21], statins [22] and newer antidiabetic drugs [23]. Although an AGE cross-link breaker has been demonstrated to reduce arterial stiffness in short-term use [24], no other study confirmed the potential beneficial effects of any specific anti-AGE or anti-AGE receptor (RAGE) drugs [25].

Nevertheless, as far as we know, no previous study in type 2 diabetes evaluated whether changes in aortic stiffness were predictive of adverse clinical outcomes. Only three previous investigations, two in individuals with end-stage renal failure under hemodialysis (26,27) and one in resistant hypertension (28), showed that aortic stiffness reduction was associated with better cardiovascular outcomes and mortality. Therefore, we aimed to explore if changes in aortic stiffness, assessed by 2 CF-PWV measurements over a 4-year period, were predictors of adverse cardiovascular outcomes and all-cause mortality in a middle-aged to elderly type 2 diabetes cohort, independent of changes in BP, HbA_1c_ and lipid levels.

## Methods

### Study overview

This was a prospective study nested within the Rio de Janeiro Type 2 Diabetes (RIO-T2D) cohort study with 417 patients with type 2 diabetes who performed a first CF-PWV measurement between 2004 and 2007 and repeated the measurement between 2009 and 2013, and were followed-up until December 2019 in the diabetes outpatient clinic of our tertiary-care University Hospital. All participants gave written informed consent, and the study protocol was approved by the local Ethics Committee. The characteristics of this cohort, the baseline procedures and the diagnostic definitions have been detailed elsewhere [11, 18, 26–28]. In brief, inclusion criteria were all adult type 2 diabetic individual up to 80 years old with either any microvascular (retinopathy, nephropathy or neuropathy) or macrovascular (coronary, cerebrovascular or peripheral artery disease) complication, or with at least two other modifiable cardiovascular risk factors. Exclusion criteria were morbid obesity (BMI ≥ 40 kg/m^2^), advanced renal failure (serum creatinine > 180 µmol/L or estimated glomerular filtration rate < 30 ml/min/1.73m^2^) or the presence of any serious concomitant disease limiting life expectancy. Specifically for this analysis, patients with peripheral arterial disease, defined by an ankle-brachial index < 0.9, or with heart failure, either detected at baseline or during the time-interval between the 2 CF-PWV measurements, were also excluded. All patients were submitted to a standard baseline protocol that included a complete clinical examination, laboratory evaluation, and CF-PWV measurement. Diagnostic criteria for diabetic chronic complications were detailed previously [11, 18, 26–28]. Briefly, coronary heart disease was diagnosed by clinical, electrocardiographic criteria, or by positive ischemic stress tests, and cerebrovascular disease was diagnosed by history and physical examination. Diabetic retinopathy was evaluated by an ophthalmologist. The diagnosis of nephropathy needed at least two albuminurias ≥30 mg/24 h or confirmed reduction of glomerular filtration rate (< 60 ml/min/1.73m^2^, or serum creatinine > 130 µmol/L). Peripheral neuropathy was ascertained by clinical examination (knee and ankle reflex activities, pinprick, temperature and vibration perception using a 128-Hz tuning fork and 10-gr monofilament pressure sensation). Neuropathy was defined as the presence of at least two of the following: symptoms, reduced pinprick, temperature and vibration perception, insensitivity to monofilament, and absent tendon reflexes. Arterial hypertension was diagnosed if mean systolic (SBP) ≥140 mmHg or diastolic BP (DBP) ≥90 mmHg on a mean of 4 BP measurements performed on two occasions at study entry or if anti-hypertensive drugs had been prescribed. For this report, office (clinic) BP was measured in the vascular laboratory immediately before each CF-PWV measurement. It was measured twice in the supine position, after at least 10 min rest, using an automatic oscillometric BP monitor Omron HEM-907 XL (Omron Healthcare, Tokyo, Japan) with a suitably sized cuff. BP considered was the mean between the two readings. Heart rate was also recorded. Mean arterial pressure (MAP) were calculated as DBP + [(SBP – DBP) / 3]. Annual relative changes in MAP between the 2 CF-PWV measurements were calculated as {[(2nd MAP – 1st MAP) / 1st MAP] * 100} / time-interval between 1st and 2nd measurements (in years). Changes in heart rate were calculated similarly. We also calculated the mean BP between the 2 CF-PWV measurements taking into account not only the 2 BPs before each CF-PWV measurement, but also all the in-between BPs measured during clinical attendances (patients had a median of 4 annual BP measurements). Laboratory evaluation included fasting glycemia, glycated hemoglobin, serum creatinine and lipids. Albuminuria and proteinuria were evaluated in two non-consecutive sterile 24-hour urine collections. We calculated the mean values of these laboratory variables obtained during the time-interval between the 2 CF-PWV measurements (all had at least 2–4 annual laboratory exams, except albuminuria which was performed once a year).

### Carotid-femoral PWV measurement

All CF-PWV measurements were performed by the same single trained independent observer, unaware of other patients’ data, using the foot-to-foot velocity method with the Complior equipment and software (Artech-Medical, Paris, France), as previously described [11, 18, 26]. Patients were in the supine position after a minimum 10 min rest at a comfortable room temperature, and all examinations were carried out in the morning, between 0900 and 1100 h, after patients had taken their morning dose of antihypertensive and antidiabetic medications. Briefly, waveforms were obtained transcutaneously by mechanotransducers over the right common carotid artery and the right femoral artery simultaneously during a minimum period of 10–15 s. The time delay (∆t) was measured between the troughs of the two waveforms, and the distance (D) covered by the waves was measured directly between the femoral and the carotid recording sites. Direct carotid-femoral distance was corrected by a factor of 0.8, as recommended [29]. CF-PWV was calculated as D (meters)/∆t (seconds). Three consecutive measurements were performed on each occasion and the mean value was used. If one of the measurements differed by > 0.5 m/s from the other measurements, it was discarded and further measurements were obtained until there were 3 CF-PWV measurements that differed by ≤ 0.5 m/s. In our laboratory, the intra-observer repeatability of CF-PWV measurement has an intra-class correlation coefficient > 0.90 and a mean relative error < 5% [18]; and the Complior equipment and procedures have been previously validated [30]. We calculated the relative annual CF-PWV changes as {[(2nd CF-PWV – 1st CF-PWV) / 1st CF-PWV] * 100} / time-interval between 1st and 2nd measurements (in % per year). CF-PWV changes were analyzed as a continuous variable and divided into quartiles (the 1st quartile with the greatest reductions and the 4th with the greatest increases in CF-PWV). We also dichotomized CF-PWV changes by considering whether CF-PWV persisted stable (relative changes between − 1% and + 1%/year), reduced (relative change <-1%/year) or increased (relative change > + 1%/year).

### Follow-up and endpoints assessment

The follow-up for this report began after the 2nd CF-PWV measurement. All patients were followed-up regularly with 2–4 annual visits until December 2019 and managed by a standardized protocol according to current guidelines. The observation period was considered as the number of months from the date of 2nd CF-PWV measurement to the date of the last clinical visit in 2019 or the first endpoint, whichever came first. The primary outcomes were the occurrence of any cardiovascular event (CVE) and all-cause mortality. Definitions of endpoints have been previously detailed [11, 27, 28]. Total CVEs included the following: fatal or non-fatal myocardial infarctions (MIs), sudden cardiac deaths, new-onset heart failure, death from progressive heart failure, any myocardial revascularization procedure, fatal or non-fatal strokes, any aortic or lower limb revascularization procedure, any amputation above the ankle, and deaths from aortic or peripheral arterial disease [11, 27, 28]. Secondary outcomes were the classic 3-point major adverse cardiovascular events (MACEs: non-fatal MIs and strokes plus all cardiovascular deaths), and cardiovascular mortality. Outcomes were adjudicated from medical records (most non-fatal and fatal in-hospital events were attended at our hospital), death certificates and interviews with attending physicians and patient families, by a standard questionnaire reviewed by two independent observers [11, 27, 28].

#### Statistical analysis

Continuous data were expressed as means and SD if normally distributed or as medians and interquartile range (IQR) if asymmetrically distributed. In initial exploratory-descriptive analysis, individuals were divided into quartiles of relative CF-PWV changes, and were compared by ANOVA (with post-hoc Bonferroni’s test), Kruskall-Wallis test or χ^2^ test, when adequate. Kaplan-Meier curves of cumulative endpoints incidence during follow-up, compared by log-rank tests, were performed for estimating different incidences of outcomes between the subgroups described previously. For assessing the independent prognostic value of CF-PWV changes parameters (continuous relative changes, its quartiles, and categorical subgroups of changes) for each outcome, time-to-event multivariate Cox analyses were undertaken. For patients with multiple events, analyses were restricted to the first event under study. Because there were evidences of non-linear associations between continuous relative CF-PWV changes and the risks of adverse outcomes (the lowest risk subgroup was not the 1st quartile but the 2nd one), we performed an extended Cox analyses with restricted natural cubic splines, which included both linear and non-linear (spline) terms, with four knots located at the 5th, 35th, 65th, and 95th percentile values of relative CF-PWV changes [31–34]. This approach allows the assessment of linear or non-linear associations, both visually and statistically, without assuming any particular functional form/contour [31–34]. In a initial exploratory analysis without defining an *a priori* reference value (i.e., with floating hazard ratios), it was observed that the lowest risk for all-cause mortality was at the − 2.4% value of relative CF-PWC changes and at -2.6% for total CVEs occurrence. Hence, in these Cox analyses with splines, we subsequently defined the reference CF-PWV change value at − 2.5%. All Cox models were adjusted for the following covariates: age, sex, first CF-PWV measurement (to account for the dependency of CF-PWV changes on their initial levels and also to control for possible regression to the mean), BMI, diabetes duration, smoking, presence of macro- and microvascular complications at baseline, anti-hypertensive and insulin treatment, mean SBP, HbA_1c_ and LDL-cholesterol levels and changes in MAP and heart rate between the first and second CF-PWV measurements. We also performed specific interaction and sensitivity analyses between CF-PWV changes with age and sex, and after excluding those individuals who died within the first 2 years after the 2nd CF-PWV measurement (to assess possible reverse causality). Results of Cox analyses were presented as adjusted hazards ratios (HRs) with their 95% confidence intervals (CIs); and a 2-tailed probability value < 0.05 was considered significant. Statistics were performed with SPSS version 19.0 (SPSS Inc, Chicago, Il., USA) and R version 3.6.0 (R Foundation for Statistical Computing, Vienna, Austria).

## Results

### Baseline characteristics and changes in CF-PWV and BPs during the measurement period

Four-hundred and seventeen individuals with T2D performed 2 CF-PWV measurements over a median time-interval of 4.2 years (IQR: 4.0-4.4 years). Mean 1st CF-PWV was 9.7 m/s (SD: 2.0; median: 9.4; IQR: 8.2–10.8 m/s); and mean 2nd CF-PWV was 10.2 m/s (SD: 2.1; median: 9.9; IQR: 8.6–11.6 m/s). There was an overall median absolute CF-PWV increase of 0.1 m/s per year, corresponding to a median relative increase of 1.1% per year. Overall, 84 individuals (20%) persisted with stable CF-PWV (relative change between − 1 and + 1% per year), 213 (51%) had increased CF-PWV (relative change > 1%) and 120 individuals (29%) had reduced CF-PWV (relative change <-1%). Table 1 outlines the baseline characteristics of all evaluated individuals and of those divided into quartiles of relative CF-PWV changes (Q1 greatest reductions; Q4 greatest increases). Patients with the greatest reductions in CF-PWV (Q1) were older and had longer diabetes duration, had a higher prevalence of arterial hypertension and were more frequently treated with ACEi/ARBs and calcium-channel blockers. They had higher BP levels at the 1st CF-PWV measurement but lower BP levels at the 2nd CF-PWV measurement, which meant that they had greater reductions in MAP between CF-PWV measurements. In contrast, individuals with the greatest CF-PWV increases (Q4) had the opposite profile: lower BP levels at the 1st CF-PWV measurement and higher BPs at the 2nd measurement; hence, greater increases in MAP between the 2 CF-PWV measurements. Otherwise, mean BP levels during the whole 4.2 year measurement period, which took into account all office BP measurements performed during this time-interval, were equivalent among quartile subgroups of CF-PWV changes. Additionally, individuals with greatest CF-PWV increases (Q4) had higher mean HbA_1c_ levels than the other subgroups. There were no differences in antidiabetic treatment among the quartile subgroups, only 17 patients were using other antihyperglycemic drugs than metformin, sulphonylureas or insulin, and none of them were using GLP-1 agonists or SGLT-2 inhibitors.

**Table 1:** Characteristics of all 417 diabetic patients evaluated and of those divided into quartiles of relative changes in aortic stiffness (Q1, greatest reductions; Q4, greatest increases)

Characteristics	All patients (n = 417)	Q1 < − 1.38% (n = 104)	Q2 − 1.38 to + 1.10% (n = 104)	Q3 + 1.11 to + 4.03% (n = 105)		Q4 > +4.03% (n = 104)		p-value
Age (years)	60.0 (9.0)	62.2 (8.7) †	58.3 (8.7)	59.9 (8.8)		59.7 (9.4)		0.017
Male sex (%)	35.7	41.3	27.9	44.8 ‡		28.8		0.018
Body mass index (kg/m^2^)	29.6 (4.7)	29.9 (4.7)	30.1 (4.5)	29.1 (4.6)		29.5 (4.9)		0.47
Smoking, current/past (%)	42.7	45.2	40.4	39.0		46.2		0.67
Diabetes duration (years)	8 (3–15)	9.5 (4–19) ‡	5.5 (2–15)	7 (3–15)		7.5 (3–14)		0.064
Chronic diabetic complications (%)								
Cerebrovascular disease	8.9	6.7	10.6	10.5		7.7		0.69
Coronary artery disease	18.0	23.1	19.2	13.3		16.3		0.30
Retinopathy	30.0	36.5	26.0	28.8		28.8		0.38
Nephropathy	26.9	26.0	27.9	25.7		27.9		0.97
Peripheral neuropathy	26.4	32.7	24.0	23.8		25.0		0.41
Diabetes treatment (%)								
Metformin	88.5	87.5	86.5	90.5		89.4		0.81
Sulfonylureas	45.3	48.1	40.4	43.8		49.0		0.57
Insulin	45.8	47.1	40.4	54.3		41.3		0.16
Other medications ^a^	4.1	3.8	3.8	4.8		3.8		0.93
Dyslipidemia (%)	88.0	84.6	86.5	90.5		90.4		0.47
Statins use (%)	75.8	73.1	74.0	76.2		79.8		0.68
Arterial hypertension (%)	85.9	94.2 †	80.8	81.0		87.5		0.015
Number of anti-hypertensive drugs	3 (1–3)	3 (2–4)	3 (1–4)	2 (1–3)		3 (1–3)		0.12
ACE inhibitors / AR blockers (%)	84.1	91.3 ‡	77.9	85.7		81.6		0.050
Diuretics (%)	67.3	73.1	65.4	61.0		69.9		0.26
Calcium channel blockers (%)	30.8	41.3	30.8	25.7		25.2		0.042
Beta-blockers (%)	48.8	53.8	48.1	43.8		49.5		0.54
Mean SBP between CF-PWV measurements (mmHg) ^b^	139 (16)	141 (14)	138 (14)	139 (16)		139 (18)		0.54
Mean DBP between CF-PWV measurements (mmHg) ^b^	77 (9)	77 (8)	76 (8)	77 (9)		78 (9)		0.69
1st CF-PVW measurement								
CF-PWV (m/s)	9.7 (2.0)	11.1 (2.3) *	9.5 (1.8)	9.5 (1.7)		8.8 (1.6) ‡		< 0.001
SBP (mmHg)	147 (23)	154 (25) ‡	147 (21)	145 (22)		143 (23)		0.002
DBP (mmHg)	80 (12)	83 (13)	80 (11)	78 (12)		78 (13)		0.025
Heart rate (bpm)	72 (12)	73 (12)	71 (13)	72 (12)		71 (12)		0.35
2nd CF-PVW measurement								
CF-PWV (m/s)	10.2 (2.1)	9.5 (2.1)	9.4 (1.8)	10.4 (1.9) *		11.3 (2.0) *		< 0.001
SBP (mmHg)	144 (24)	139 (22)	139 (21)	146 (23)		152 (28) *		< 0.001
DBP (mmHg)	76 (14)	72 (12)	73 (12)	77 (17)		81 (14) *		< 0.001
Heart rate (bpm)	69 (12)	68 (11)	68 (13)	69 (12)		71 (12)		0.26
Time-interval between CF-PWV measurements (years)	4.2 (0.6)	4.2 (0.5)	4.3 (0.5)	4.2 (0.6)		4.2 (0.6)		0.45
Relative CF-PWV change (% per year)	+ 1.1 (− 1.4 to +4.0)	− 2.8 (− 4.4 to − 2.0) *	− 0.2 (− 0.8 to +0.4)	+ 2.1 (+ 1.5 to +3.0) *		+ 5.9 (+ 4.9 to +8.4) *		< 0.001
Relative MAP change (% per year)	-0.9 (-4.0 - +2.4)	-3.4 (-6.3 - +0.6) ‡	-1.7 (-4.2 - +1.4)	+ 0.2 (− 2.9 to +2.8) ‡		+ 1.8 (− 1.7 to +3.3) *		< 0.001
Relative heart rate change (% per year)	− 0.9 (− 3.4 to +1.1)	− 1.4 (− 3.8 to +0.7)	− 0.8 (− 2.9 to +1.8)	− 1.0 (− 3.6 to +0.6)		-0.5 (− 2.6 to +2.6)		0.045
Laboratory variables ^b^								
Fasting glycemia (mmol/l)	8.0 (2.8)	7.8 (2.7)	7.9 (2.9)	8.2 (2.8)		8.0 (2.8)		0.73
HbA_1c_ (%)	7.6 (1.3)	7.4 (1.1)	7.5 (1.3)	7.7 (1.3)		7.9 (1.5) ‡		0.008
(mmol/mol)	60 (14.2)	57 (12.0)	58 (14.2)	61 (14.2)		63 (16.4)		
Triacylglycerol (mmol/l)	1.8 (1.2)	1.8 (1.2)	1.9 (1.4)	1.9 (1.2)		1.9 (1.1)		0.90
HDL-cholesterol (mmol/l)	1.1 (0.3)	1.1 (0.3)	1.1 (0.3)	1.1 (0.3)		1.1 (0.3)		0.98
LDL-cholesterol (mmol/l)	2.6 (0.6)	2.5 (0.6)	2.6 (0.6)	2.7 (0.7)		2.6 (0.7)		0.44
eGFR (ml/min/1.73m^2^)	72 (18)	71 (17)	73 (20)	75 (17)		70 (17)		0.24
Albuminuria (mg/24 h)	16 (8–44)	15 (8–39)	17 (9–46)	15 (9–33)		21 (8–73)		0.61
Outcomes incidence, absolute number (incidence rate per 1000 person-years of follow-up)								
Total cardiovascular events	101 (33.6)	32 (43.6) ‡	17 (20.8)	30 (41.7) ‡		22 (30.0)		0.056
Major adverse cardiovascular events	85 (27.8)	28 (37.1) †	13 (15.4)	24 (33.0) ‡		20 (27.1)		0.051
All-cause mortality	135 (42.7)	35 (44.9) †	17 (19.7)	40 (53.5) *		43 (55.8) *		0.001
Cardiovascular mortality	64 (20.3)	23 (29.5) †	8 (9.3)	19 (25.4) ‡		14 (18.2)		0.021

Values are proportions, and means (standard deviations) or medians (interquartile range), except for outcomes incidences

Baseline characteristics were obtained at the 2nd CF-PWV measurement, except when indicated

Outcomes assessment began after the 2nd CF-PWV measurement

*ACE* angiotensin-converting enzyme,* AR* angiotensin II receptor,* SBP* systolic blood pressure,* DBP* diastolic blood pressure,* CF-PWV* carotid-femoral pulse wave velocity,* MAP* mean arterial pressure, *HbA*_1c_ glycated hemoglobin,* HDL* high-density lipoprotein,* LDL* low-density lipoprotein,* eGFR* estimated glomerular filtration rate

^a^ 17 patients were using other antidiabetic medications: 12 were using DPP-4 inhibitors and 5 were using thiazolidinediones, none were using GLP-1 agonists or SGLT-2 inhibitors

^b^ Mean values obtained in the time-interval between the 1st and 2nd CF-PWV measurements

Post-hoc comparisons among quartile subgroups were performed against the reference 2nd quartile subgroup with Bonferroni’s correction for multiple comparisons: *p < 0.001; †p < 0.01; ‡p < 0.05

### Follow-up and incidence of adverse outcomes

**Fig. 1:**
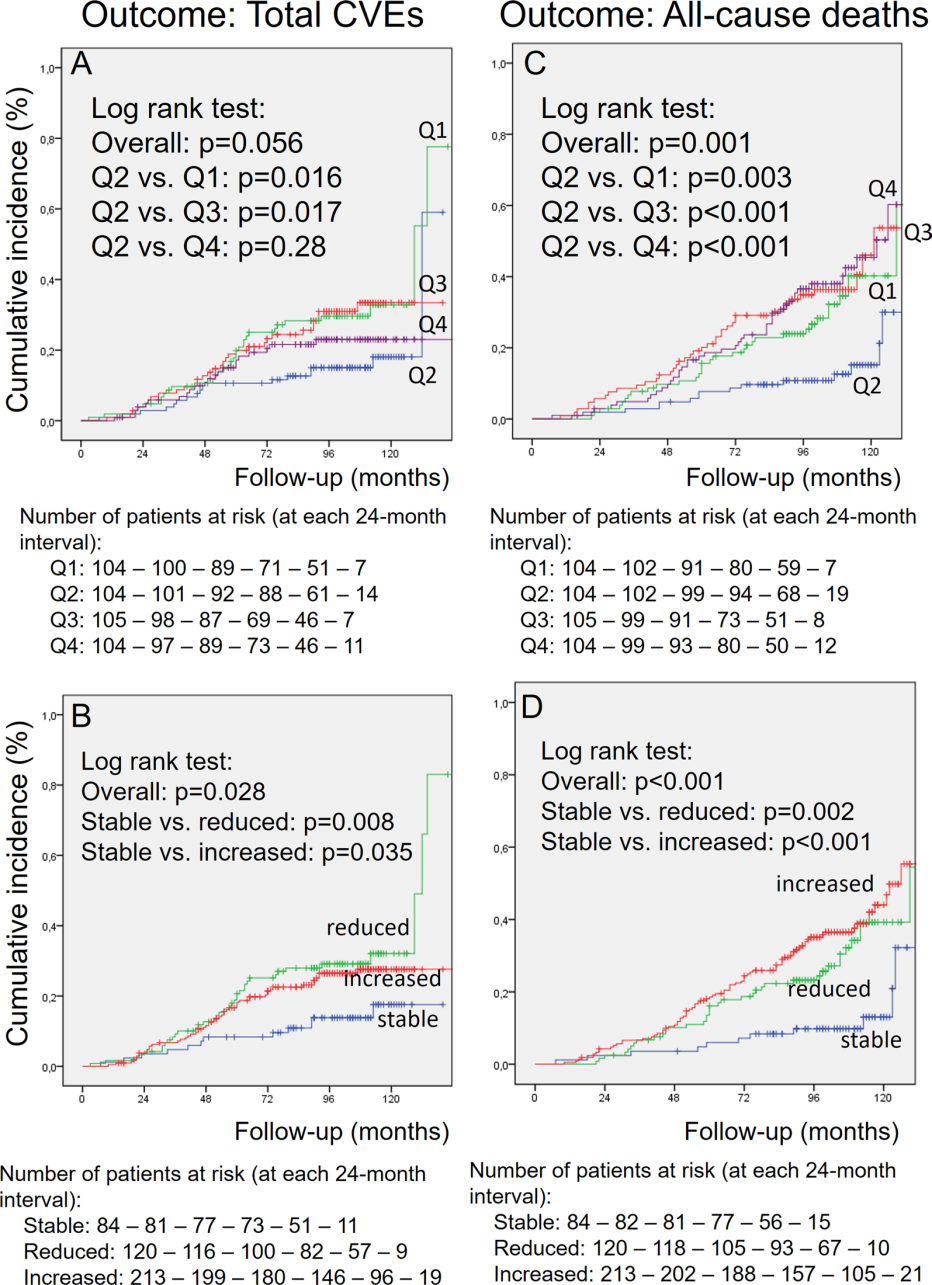
Kaplan-Meier curves of cumulative incidences of total cardiovascular events (CVEs, left panels **A** and **B**) and of all-cause deaths (right panels **C** and **D**) in individuals with type 2 diabetes divided according to quartiles of changes in carotid-femoral pulse wave velocity (CF-PWV, upper panels **A** and **C**) and to having persisted with stable, increased or reduced CF-PWV (bottom panels **B** and **D**)

**Fig. 2:**
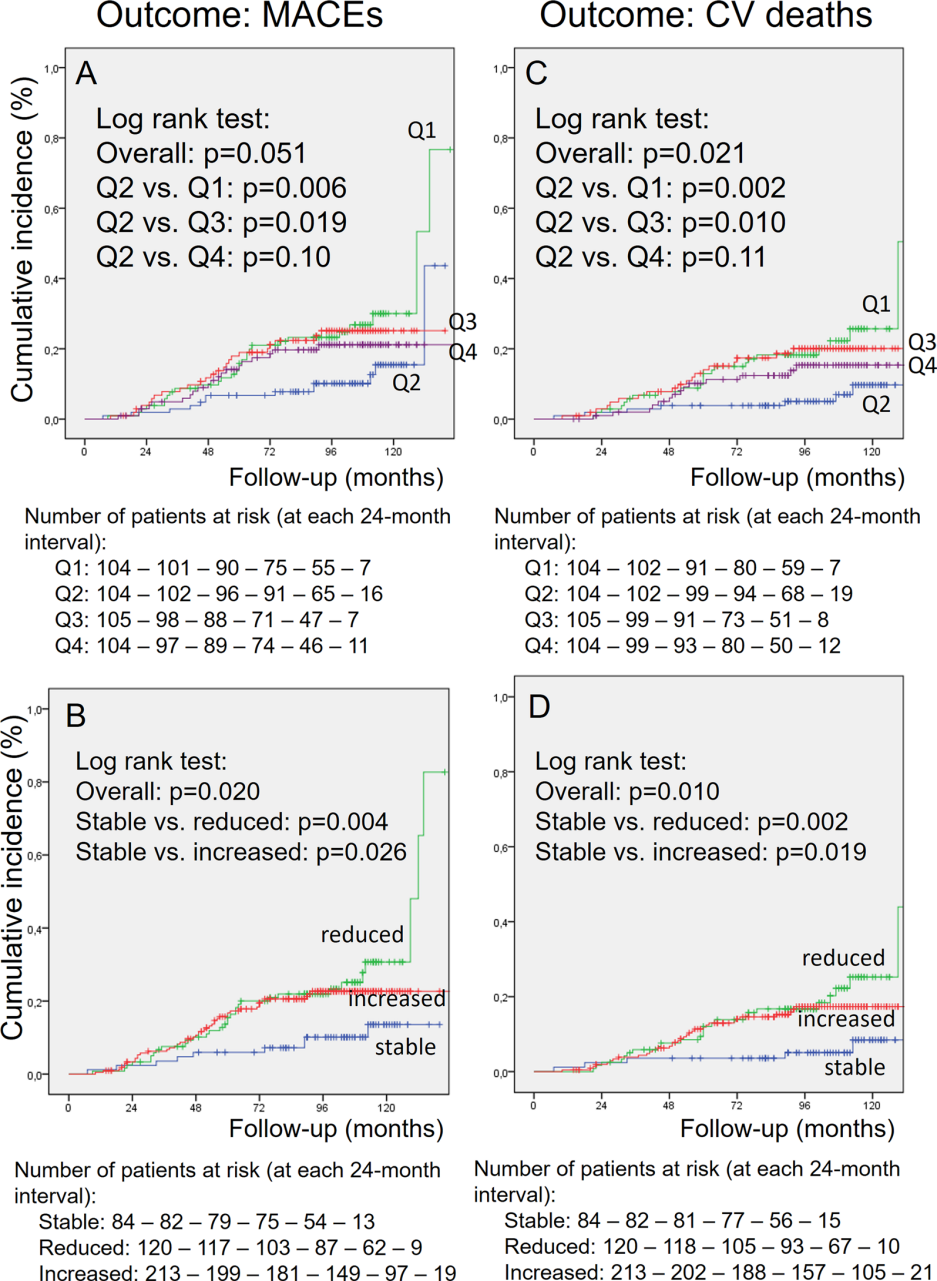
Kaplan-Meier curves of cumulative incidences of major adverse cardiovascular events (MACEs, left panels **A** and **B**) and of cardiovascular deaths (right panels **C** and **D**) in individuals with type 2 diabetes divided according to quartiles of changes in carotid-femoral pulse wave velocity (CF-PWV, upper panels **A** and **C**) and to having persisted with stable, increased or reduced CF-PWV (bottom panels **B** and **D**)

After the CF-PWV measurement period, patients were further followed-up for a median of 8.2 years (IQR: 6.3–9.2 years, maximum 11 years), which corresponded to 3,160 person-years (PY) of follow-up, over which there were 101 total CVEs (incidence rate: 33.6 per 1,000 PY), 85 MACEs (27.8 per 1,000 PY), and 135 all-cause deaths (42.7 per 1,000 PY), 64 from cardiovascular causes. Table 1 (bottom) shows that the incidence rates of all adverse outcomes were lowest in the 2nd quartile subgroup (Q2) and significantly increased in the 1st and 3rd quartile subgroups of CF-PWV changes, which indicated probable non-linear associations between CF-PWV changes and risks of adverse outcomes. The lowest incidence of adverse outcomes in the Q2 subgroup was confirmed in Kaplan-Meier curves shown on Figs. 1 and 2 (upper panels). Figures 1 and 2 (lower panels) also demonstrated that the stable CF-PWV subgroup also had the lowest cumulative incidences of adverse outcomes in contrast to those who either had greater reductions or increases in CF-PWV.

### Risks associated with changes in CF-PWV

**Fig. 3:**
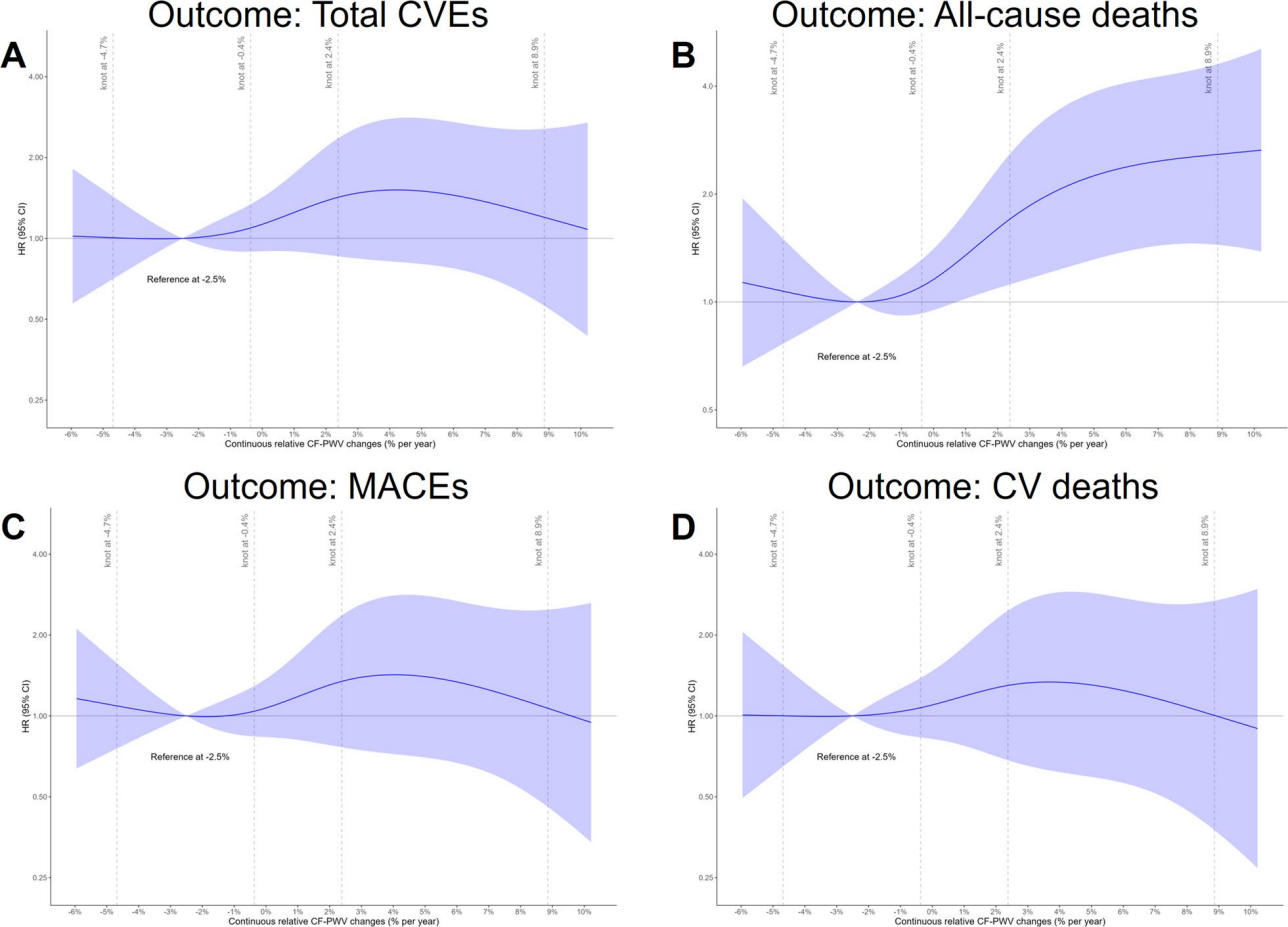
Adjusted risks associated with continuous relative annual carotid-femoral pulse wave velocity (CF-PWV) changes for total cardiovascular events (CVEs, panel **A**), all-cause mortality (panel **B**), major adverse cardiovascular events (MACEs, panel C) and cardiovascular mortality (panel D), modeled by extended Cox analyses with splines (knots at the 5th, 35th, 65th, and 95th percentile values, and reference value at − 2.5%/year change, the lowest risk nadir point). All analyses were adjusted for age, sex, first CF-PWV measurement, BMI, diabetes duration, smoking, presence of macro- and microvascular complications at baseline, anti-hypertensive and insulin treatment, mean SBP, HbA_1c_ and LDL-cholesterol levels and changes in MAP and heart rate between the first and second CF-PWV measurements

Figure 3 presents graphically the adjusted risks associated with continuous relative CF-PWV changes modeled by Cox regressions with cubic splines to account for non-linear relationships. The lowest risk nadir point was estimated at -2.5% per year CF-PWV change, with significantly increased risks to the right of this nadir point (i.e., at greater increases in CF-PWV, beginning at + 1.0%) for all-cause mortality, but without statistical significance for the other outcomes. To the left of the nadir point of CF-PWV changes (i.e., at greater reductions than − 2.5%), there were slight non-significant excess risks for all-cause mortality and MACEs outcomes.

Table 2 outlines the adjusted risks associated with CF-PWV changes categorized into quartiles and also as stable/reduction/increase subgroups. Patients in the Q1 (greatest CF-PWV reductions) had significantly excess risks of all-cause and cardiovascular mortality, with HRs between 2.0 and 2.7, and a borderline 1.9-fold increased risk of MACEs; whereas patients in Q3 had significantly higher risks of all outcomes, with HRs ranging from 2.0 to 3.2, in relation to the reference Q2 subgroup. Patients in the Q4 (greatest increases in CF-PWV) had significantly higher risks of all-cause and cardiovascular mortality, but not of total CVEs or MACEs. When categorized as stable/reduction/increase subgroups, patients with greater CF-PWV reductions had significantly higher risks of MACEs, all-cause and cardiovascular mortality, with HRs from 2.2 to 3.3; whereas those with greater CF-PWV increases had significantly excess risks of all outcomes, with adjusted HRs ranging from 1.9 to 3.4, in relation to the reference category who remained with stable CF-PWV. No evidence of interaction of CF-PWV changes with age or sex was found (all p-for-interaction values > 0.30), signifying that the risks associated with aortic stiffness changes were similar between older/younger individuals and men/women. Excluding the individuals who died within the first 2 years of follow-up also did not change any of the results, indicating that there was no reverse causality between CF-PWV changes and adverse outcomes.

**Table 2:** Risks of adverse outcomes associated with categorical changes in aortic stiffness, assessed by multivariable Cox regressions

CF-PWV changes	Total CVEs (n = 101)		MACEs (n = 85)		All-cause mortality (n = 135)		Cardiovascular mortality (n = 64)	
	HR (95% CI)	p-value	HR (95% CI)	p-value	HR (95% CI)	p-value	HR (95% CI)	p-value
Quartiles of annual relative change								
Q1 ( < − 1.38% per year)	1.48 (0.78–2.82)	0.23	1.94 (0.96–3.93)	0.067	1.98 (1.07–3.65)	0.029	2.71 (1.15–6.40)	0.023
Q2 (− 1.38 to + 1.10% per year)	1.0 (REF)	–	1.0 (REF)	–	1.0 (REF)	–	1.0 (REF)	–
Q3 (+ 1.11 to + 4.03% per year)	1.96 (1.06–3.64)	0.032	2.32 (1.10–4.51)	0.025	3.16 (1.75–5.70)	< 0.001	3.15 (1.33–7.43)	0.009
Q4 ( > + 4.03% per year)	1.45 (0.74–2.83)	0.28	1.71 (0.81–3.63)	0.16	3.19 (1.75–5.82)	< 0.001	2.00 (0.80-5.00)	0.14
Relative annual change								
Reduced (< − 1% per year)	1.73 (0.86–3.48)	0.12	2.32 (1.07–5.04)	0.034	2.15 (1.10–4.20)	0.025	3.31 (1.22–8.99)	0.019
Stable (− 1% to + 1% per year)	1.0 (REF)	–	1.0 (REF)	–	1.0 (REF)	–	1.0 (REF)	–
Increased ( > + 1% per year)	1.90 (1.00-3.60)	0.050	2.29 (1.09–4.81)	0.028	3.39 (1.81–6.35)	< 0.001	3.23 (1.23–8.49)	0.017

Hazard ratios were estimated from Cox analyses adjusted for the following covariates: age, sex, first CF-PWV measurement, BMI, diabetes duration, smoking, presence of macro- and microvascular complications at baseline, anti-hypertensive and insulin treatment, mean SBP, HbA_1c_ and LDL-cholesterol levels and changes in MAP and heart rate between the first and second CF-PWV measurements

*CVEs* cardiovascular events,* MACEs* major adverse cardiovascular events,* CF-PWV* carotid-femoral pulse wave velocity,* HR* hazard ratio,* CI* confidence interval,* Q1 to Q4* quartile subgroups from the greatest reduction (Q1) to the greatest increase (Q4)

## Discussion

To the best of our knowledge, this is the first longitudinal study that evaluated the prognostic importance of aortic stiffness changes, assessed by its gold-standard CF-PWV measurement, in patients with type 2 diabetes. We demonstrated that a progressively increasing aortic stiffness over a 4-year period was associated with significantly higher risks of adverse cardiovascular outcomes and of all-cause mortality, independent of classic risk factors and of other factors that might influence CF-PWV changes, such as changes in BP and heart rate and mean BP, HbA_1c_ and lipid levels between the 2 CF-PWV measurements. This was observed both in analyses with continuous and categorical CF-PWV changes. Otherwise, we also evidenced that extreme CF-PWV reductions, possibly greater than − 2.5% to -3.0% per year, might be deleterious. However, this observation may be faced with caution, as this was evidenced only in analyses of categorical CF-PWV changes, but not supported in the spline analyses with continuous CF-PWV changes. Overall, our study suggested that monitoring aortic stiffness changes overtime, aiming to prevent its progression, may be relevant to reduce cardiovascular morbidity and mortality burden in individuals with type 2 diabetes.

There were only 3 previous studies that evaluated changes in aortic stiffness as predictors of adverse outcomes, two of them in patients with end-stage renal failure undergoing dialysis [35, 36] and the other in patients with resistant hypertension [37]. They all agreed that preventing progression/increase in aortic stiffness was protective for cardiovascular outcomes [36, 37] and all-cause mortality [35, 37]; and none of them detected any signal of non-linear J-curve associations between CF-PWV changes and outcomes. Furthermore, most of the previous studies that evaluated the prognostic importance of a single CF-PWV measurement also reported linear dose-dependent associations between aortic stiffness and adverse outcomes [10], including those that evaluated exclusively patients with diabetes [11, 12, 38]. As far as we know, only one previous study [39], with 3034 elderly individuals without CVDs from the Atherosclerosis Risk in Communities (ARIC) cohort, reported a J-curve association between a single CF-PWV measurement and incident CVD, with hazard ratios of 1.8 and 2.0, respectively in the 4th and 1st quartile subgroups in relation to 2nd quartile subgroup. However, no spline analysis with continuous CF-PWV was performed to corroborate the possible non-linear association found in categorical analyses with quartile subgroups [39]. The reasons for these possible J-curve associations were unclear. In our study, patients in the 1st quartile subgroup of CF-PWV changes (i.e., with greatest CF-PWV reductions) were older, had longer diabetes duration and higher prevalence of arterial hypertension and, albeit they used more anti-hypertensive medications, they had higher BP levels and higher CF-PWV at the first measurement. Although we had adjusted all multivariable analyses for these covariates, some residual confounding may have still remained, which might have contributed to the increased risks observed in the subgroup with greatest CF-PWV reduction. Another possible explanation was some reverse causation between CF-PWV changes and adverse outcomes; that is, it may be the nearby event, particularly death, that might have driven the greater CF-PWV reduction, not the opposite. However, when we excluded from analysis those individuals who had any event during the first 2 years of follow-up, the 1st quartile subgroup still had higher risks than the reference 2nd quartile subgroup, not supporting the occurrence of reverse causation. Moreover, as pointed out before, there were no increased risks at the extreme of CF-PWV reduction in the Cox analyses with splines for any of the outcomes. Therefore, we believe that the observation that greater aortic stiffness reductions might be hazardous should be faced as hypothesis-generating and not as definite evidence, and it shall be examined in other larger cohorts of individuals with diabetes.

Aortic stiffness may be an integrate indicator not only of the effects of ageing and genetic background, but also of the cumulative damage of cardiovascular risk factors on the arterial wall overtime [7–9]. Current evidences [8, 15, 40] suggest that only part of aortic stiffness could be reduced through normalization of blood pressure by pharmacologic treatment; additional decrease of aortic stiffness would depend on long-term arterial remodeling, including reduction in collagen density and reorganization of arterial wall components. Particularly in diabetes, aortic stiffness may be accelerated by long-term hyperglycemia and formation of AGEs on the arterial wall, with loss of elastin and increased collagen cross-links [41]. Medications that are in current use for diabetes and hypertension may have potential anti-AGE or -RAGE effects. However, it is probable that the primary effects of antidiabetic, antihypertensive and statin drugs are more important than their pleiotropic secondary AGE or RAGE opposing effects [25]. The rationale of maintaining low stable aortic stiffness shall still be based on long-term optimal metabolic and BP control by pharmacological and non-pharmacological therapies [7, 8, 19, 20, 22, 23].

This study has limitations that shall be noted. First, this is an observational cohort study on the prognostic value of changes in CF-PWV and, as such, no direct inference can be made regarding cause-and-effect relationships or physiopathological mechanisms. Second, as already pointed out, as in any cohort study, some residual confounding cannot be ruled out. Third, as in any study with serial measurements of a biological variable, the possibility of influence of the ‘regression-to-the-mean’ phenomenon and its associated ‘regression dilution bias’ should be acknowledged. However, as previously demonstrated [18], the overall ‘regression-to-the mean’ effect in these two CF-PWV measurements was small. Finally, our cohort was initiated in 2004, and the use of the new antidiabetic medications with cardiovascular protection effects was very rare. Hence, our findings might not be generalized to individuals with diabetes using these new drugs. Otherwise, currently, most people with diabetes are still using the traditional drug treatments with metformin, sulphonylureas and insulin, as in our cohort. Furthermore, this cohort enrolled mainly middle-aged to elderly individuals with long-standing type 2 diabetes followed-up in a tertiary-care university hospital. Hence, our results might also not be generalized to younger individuals with recent onset diabetes or to less severe ones at primary care management. On the other hand, strengths of this study include a well-characterized cohort of individuals with type 2 diabetes actively followed-up under a standardized management, and the multivariable statistical adjustment for several potential confounders that may have affected CF-PWV changes [18].

## Conclusions

This prospective study in individuals with type 2 diabetes provides evidence that changes in aortic stiffness, assessed by 2 CF-PWV measurements over a 4-year interval, are independent risk markers for adverse cardiovascular outcomes and mortality. Patients who had increases in CF-PWV had significantly worse cardiovascular and mortality prognoses than those who remained with stable or moderately-reduced CF-PWV, although extreme CF-PWV reductions might be deleterious. Therefore, depending on confirmation from larger studies, monitoring aortic stiffness changes by serial CF-PWV assessments, aiming to prevent its progression, may be relevant for reducing cardiovascular and mortality risks in patients with type 2 diabetes. Whether a therapeutic strategy based on serial CF-PWV measurements may be recommended in the clinical management of diabetes, this shall be examined in future studies.

## Data Availability

The Rio de Janeiro Type 2 Diabetes Cohort Study is an on-going study, and its dataset is not publicly available due to individual privacy of the participants. However, it may be available from the corresponding author on reasonable request.

## Abbreviations

AGEs: Advanced glycation end-products
BMI: Body mass index
BP: Blood pressure
CF-PWV: Carotid-femoral pulse wave velocity
CI: Confidence interval
CVEs: Cardiovascular events
DBP: Diastolic blood pressure
HbA1c: Glycated hemoglobin
HR: Hazard ratio
MACEs: Major adverse cardiovascular events
MAP: Mean arterial pressure
RAGE: Advanced glycation end-products receptor
SBP: Systolic blood pressure
